# Transformation behaviour of salts composed of calcium ions and phosphate esters with different linear alkyl chain structures in a simulated body fluid modified with alkaline phosphatase

**DOI:** 10.1080/14686996.2022.2074801

**Published:** 2022-05-30

**Authors:** Taishi Yokoi, Akiyoshi Mio, Jin Nakamura, Ayae Sugawara-Narutaki, Masakazu Kawashita, Chikara Ohtsuki

**Affiliations:** a Institute of Biomaterials and Bioengineering, Tokyo Medical and Dental University (TMDU), Tokyo, Japan; b Graduate School of Engineering, Nagoya University, Nagoya, Japan

**Keywords:** ceramic biomaterials, phosphate esters, Alkaline phosphatase, simulated body fluid, calcium phosphates, transformation

## Abstract

Ceramic biomaterials have been used for the treatment of bone defects and have stimulated intense research on such materials. We have previously reported that a salt composed of calcium ions and a phosphate ester (SCPE) transformed into hydroxyapatite (HAp) in a simulated body fluid (SBF) modified with alkaline phosphatase (ALP), and proposed SCPEs as a new category of ceramic biomaterials, namely bioresponsive ceramics. However, the factors that affect the transformation of SCPEs to HAp in the SBF remained unclear. Therefore, in this study, we investigated the behaviour of calcium salts of methyl phosphate (CaMeP), ethyl phosphate (CaEtP), butyl phosphate (CaBuP), and dodecyl phosphate (CaDoP) in SBF with and without ALP modification. For the standard SBF, an X-ray diffraction (XRD) analysis indicated that these SCPEs did not readily transform into calcium phosphate. However, CaMeP, CaEtP, and CaBuP were transformed into HAp and octacalcium phosphate in the SBF modified with ALP; therefore, these SCPEs can be categorised as bioresponsive ceramics. Although CaDoP did not exhibit a sufficient response to ALP to be detected by XRD, it is likely to be a bioresponsive ceramic based on the results of morphological observations. The transformation rate for the SCPEs decreased with increasing size of the linear alkyl group of the phosphate esters. The rate-determining steps for the transformation reaction of the SCPEs were changed from the dissolution of the SCPEs to the hydrolysis of the phosphate esters with increasing size of the phosphate ester alkyl groups. These findings contribute to designing novel bioresponsive ceramic biomaterials.

## GRAPHICAL ABSTRACT

**Figure:**
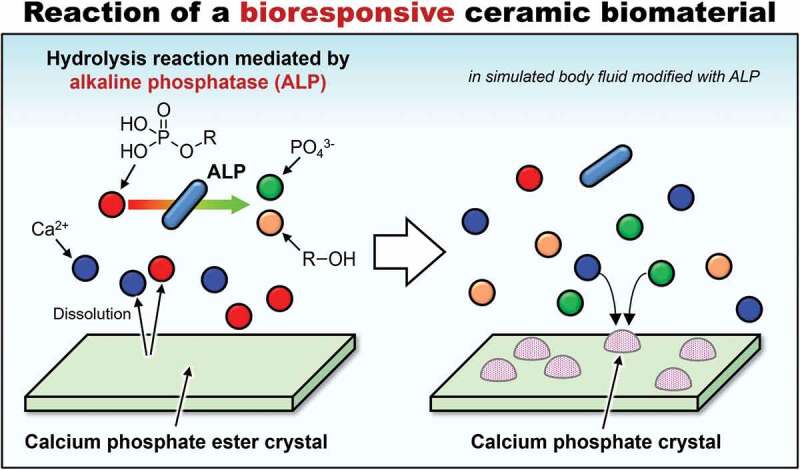

## 1. Introduction

Biomaterials research has grown to become one of the major research areas in the materials field. One of the reasons for this is the diversity of the materials used. Polymers, ceramics, metals, and their composites are used in a variety of medical materials [1–3]. Precisely designed and synthesized polymers have made a significant contribution to the development of advanced medical technologies, especially regenerative medicine [4–8]. On the other hand, ceramic and metallic materials are mainly studied as structural materials to repair bones [9–13] and joints [14–18], respectively. These structural materials are used for a long time *in vivo*; therefore, properties such as corrosion resistance, wear resistance and fatigue strength are very important. However, when considering the design of next-generation biomaterials, it is necessary to design materials based on new ideas that are not bound by conventional frameworks.

In terms of material science, ceramics are more similar to metals than to polymers. However, we consider that learning from organic chemistry and polymer chemistry is worthwhile, and is a shorter way to design and develop highly functional ceramic biomaterials. Functional metal-organic frameworks [19–23], sol-gel derived inorganic-organic hybrid materials [24–28] and organically modified inorganic layered compounds [29–33] are typical examples of this. If we can not only partially incorporate organic components into ceramics as in these cases, but also incorporate highly designed polymeric materials, then this would enable the design of even more functional ceramic biomaterials.

We are focused on materials that respond to enzymes present in the human body. Polymers that express their functions in response to enzymes in the body have been reported to date, such as anticancer-drug-loaded poly(amidoamine) dendrimers [34] and polytyrosine nanoparticles [35], as well as doxorubicin-loaded, triphenylphosphine-attached, hyaluronic acid-capped mesoporous silica nanoparticles [36]. Not only polymers, but certain ceramics have been known to react with enzymes. It has been reported that hydroxyapatite (HAp, (Ca_10_(PO_4_)_6_(OH)_2_), which has been commonly used as artificial bone, can be synthesized *in vitro* by the reaction of calcium ions and phosphate ions generated by hydrolysation of phosphate esters mediated by alkaline phosphatase (ALP) [37–41]. ALP is present in human serum; therefore, salts of calcium ions and phosphate esters (SCPEs) are good candidates as novel ceramic biomaterials, especially bone-repairing materials that respond to ALP. Based on this idea, in 2020, our research group demonstrated that calcium phenyl phosphate, a type of SCPE, transformed into HAp in a simulated body fluid (SBF) modified with ALP, although the pH of the SBF was 7.4, which was outside the optimal pH range for ALP [42]. We consider that such ceramics will become the fourth category of bioresponsive ceramics, after bioinert, bioactive, and bioabsorbable ceramics.

**Figure 1.**
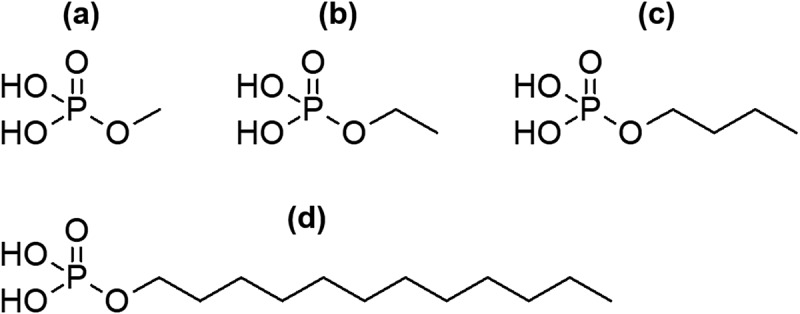
Molecular structures of (a) methyl phosphate, (b) ethyl phosphate, (c) butyl phosphate and (d) dodecyl phosphate.

SCPEs are bioresponsive ceramics that are likely absorbed by the action of ALP *in vivo*. In SCPEs that are intended to be gradually absorbed and replaced by bone after implantation in the body, control of the absorption rate and the transformation rate for SCPEs into HAp after implantation in the body is very important. However, the factors that affect the transformation rate for SCPEs into HAp in the presence of ALP, such as the solubility of SCPEs and the hydrolysis rate for phosphate esters mediated by ALP, have not yet been clarified. If we can identify the factors that control the rate at which SCPEs are transformed into HAp by ALP, then this would allow for the design of SCPEs that transform into HAp at an optimal rate *in vivo*. To determine whether the alkyl group of phosphate esters is a factor that controls the rate of HAp formation from SCPEs, methyl phosphate, ethyl phosphate, butyl phosphate, and dodecyl phosphate (Figure 1) were selected as phosphate esters with linear alkyl groups for comparison, and their calcium salts were soaked in ALP-modified SBF to compare their HAp transformation behaviour.

## 2. Experiments

### 2.1. Synthesis and characterization of the SCPEs

Appropriate amounts of calcium chloride (CaCl_2_, Nacalai Tesque Inc., Kyoto, Japan) were dissolved in ultrapure water and the pH of the solution was adjusted to 10 with 28 mass% ammonia water (NH_3_, Wako Pure Chemical Industries, Ltd., Osaka, Japan) at 37°C to prepare 50 mol·m^−3^ (mM) and 200 mM of CaCl_2_ solutions.

200 mM of methyl phosphate, ethyl phosphate, and butyl phosphate solutions with pH 10 at 37°C were prepared by dissolving these phosphate esters in ultrapure water. The phosphate esters were purchased from Tokyo Chemical Industry Co., Ltd., Tokyo, Japan and were mixture of mono and diesters. The monoester contents of methyl phosphate, ethyl phosphate, and butyl phosphate were 50.0–55.0 mass%, 35.0–47.0 mass%, and 34.0–45.0 mass%, respectively. Ammonia water was used for pH adjustment. A 200 mM CaCl_2_ solution (250 cm^3^) and a 200 mM phosphate ester (methyl- ethyl- and butylesters) aqueous solution were mixed at 37°C with stirring for 2 h. At this time, pH values of the reaction solution containing methyl- ethyl- and butylesters were 9.8, 9.8, and 9.8, respectively. The formed powders composed of calcium and the phosphate esters were then obtained by suction filtration and washing, followed by drying for one day at 40°C.

Additionally, a 50 mM dodecyl phosphate solution with pH 10 at 37°C was prepared by dissolving dodecyl phosphate (Wako Pure Chemical Industries, Ltd.) in ultrapure water. Ammonia water was used for the pH adjustment. A 50 mM CaCl_2_ aqueous solution (250 cm^3^) and a 50 mM dodecyl phosphate aqueous solution (250 cm^3^) were mixed at 37°C with stirring for 2 h. At this time, the pH of the reaction solution was 9.8. The formed calcium dodecyl phosphate powder was then obtained by suction filtration and washing, followed by drying for one day at 40°C. The reason for changing the synthesis conditions, that is, a decrease in the solution concentrations of calcium dodecyl phosphate from those of the other calcium phosphate esters synthesis was that 200 mM of dodecyl phosphate solution with pH 10 could not be prepared at 37°C due to the low solubility of dodecyl phosphate. Hereafter, the synthesized SCPEs using methyl phosphate, ethyl phosphate, butyl phosphate, and dodecyl phosphate are denoted as CaMeP, CaEtP, CaBuP, and CaDoP, respectively.

The crystalline phases of the synthesized samples were characterized using powder X-ray diffraction (XRD; RINT2000, Rigaku Co., Ltd., Tokyo, Japan) with Cu Kα radiation. The chemical structures of the samples were characterized using Fourier transform infrared spectroscopy (FTIR; FT/IR-610, JASCO Corp., Tokyo, Japan) with the KBr tablet method. The mass ratio of KBr powder: sample was 100:1. The KBr powder used was FTIR absorption measurement grade (Wako Pure Chemical Industries, Ltd.). The morphology of the samples was observed using scanning electron microscopy (SEM; JEM-5600, JEOL Ltd., Tokyo, Japan) after thin gold film coating.

The compositions, that is, Ca/P molar ratio, of the synthesized SCPEs were measured using inductively coupled plasma atomic emission spectroscopy (ICP-AES; Optima2000DV, PerkinElmer Japan Co., Ltd., Kanagawa, Japan) after dissolving the samples in dilute nitric acid. The solubility of the synthesized SCPEs was also measured; an excess amount of the synthesized SCPEs was dispersed in 25 cm^3^ of ultrapure water and kept at 37°C for one day, and then the Ca and P concentrations of the solution was measured using ICP-AES.

### 2.2. Behaviour of the synthesized SCPEs in SBF containing ALP

The SBF, that is, the Kokubo solution, was prepared as previously reported [42]. The preparation procedures for the SBF are described in the supplementary information. A polystyrene container was filled with 25 cm^3^ of SBF, and 1.0 mmol of the synthesized SCPEs was soaked in the SBF. The formula weight of the synthesized SCPEs was calculated assuming that the SCPEs are composed of calcium ions and phosphate ester in a 1:1 molar ratio. The ALP (from bovine intestinal mucosa, Sigma-Aldrich Japan Co., Tokyo, Japan) was added daily to the SBF in 1.25 units and kept in an incubator at 36.5°C for 1, 3 and 7 days. The sample powder was then collected by filtration and dried at 40°C. ALP was added to the SBF at daily intervals due to its gradual deactivation. The ALP concentration in SBF was approximately 50 units·dm^−3^, which is in the range of the standard concentration of ALP in human plasma (20–140 units·dm^−3^) [43]. As a control experiment, the synthesized SCPEs were also soaked in SBF without ALP to investigate their reaction behavior.

The changes in the crystalline phases and morphologies of the samples by soaking in the SBF were characterized using XRD and SEM. The changes in concentrations of Ca and P in the SBF by soaking of the samples were measured using ICP-AES. Changes in the pH of the SBF by soaking of the samples were also measured using a glass-electrode-type pH meter (D-53, Horiba Ltd., Kyoto, Japan).

## 3. Results

### 3.1. Characterization of the as-synthesized SCPEs

Table 1 summarizes the Ca/P molar ratios and the experimentally determined solubility product for the samples. The Ca/P ratios for all samples were almost 1, that is, all samples were composed of Ca ions: phosphate ester in a composition almost equal to 1:1. The solubility products for the samples were thus roughly calculated as the product of the Ca and P concentrations. The solubility products based on this assumption for CaMeP, CaEtP, CaBuP, and CaDoP were 9.1 × 10^−5^, 9.4 × 10^−5^, 9.5 × 10^−6^, and 1.4 × 10^−9^ mol^2^·dm^−6^, respectively. Although, the solubility of CaMeP and CaEtP was almost the same, when the alkyl group of the phosphate ester was a butoxy group, the solubility of the calcium phosphate ester (CaBuP) became smaller. In addition, when the alkyl group of the phosphate ester was a dodecyl group, the solubility of the calcium phosphate ester (CaDoP) became extremely small.

**Table 1.** Ca/p molar ratios and experimentally determined solubility products of CaMeP, CaEtP, CaBuP, and CaDoP

Samples	Ca/P molar ratio	Solubility product* (mol^2^·dm^–6^)
CaMeP	1.06	9.1 × 10^−5^
CaEtP	0.99	9.4 × 10^−5^
CaBuP	0.97	9.5 × 10^−6^
CaDoP	1.04	1.4 × 10^−9^

*Note that the solubility product is the product of the Ca and P concentrations from solutions in which CaMeP, CaEtP, CaBuP, and CaDoP were soaked.

**Figure 2.**
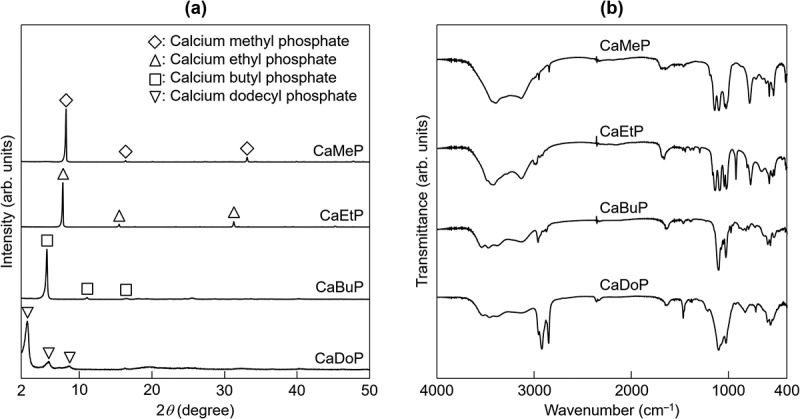
(a) XRD patterns and (b) FTIR spectra for CaMeP, CaEtP, CaBuP and CaDoP.

Figure 2(a) shows XRD patterns for the synthesized SCPEs. XRD patterns for calcium methyl phosphate and calcium ethyl phosphate have not been reported. However, the crystalline phases of CaMeP and CaEtP were most likely attributable to calcium methyl phosphate and calcium ethyl phosphate, respectively, based on the results of compositional analysis and FTIR measurements, which are discussed later. CaBuP and CaDoP were attributed to calcium butyl phosphate and calcium dodecyl phosphate, respectively, in accordance with the literature [44,45]. Figure 2(b) shows FTIR spectra of the synthesized SCPEs. The absorption peaks detected at 2980 to 2921 cm^−1^ and 2873 to 2843 cm^−1^ were derived from C-H stretching [46]. In addition, the absorption peaks detected at 1105 to 1095 cm^−1^ were assignable to P-O stretching, estimated based on the absorption peak derived from phosphate ions in a calcium orthophosphate compound [47]. The absorption peaks associated with P-O-C stretching were detected at 1027 cm^−1^ [45].

From the results of the compositional analysis, XRD and FTIR, the synthesized SCPEs were most likely a single phase. Therefore, the next section shows the results for the reaction behaviour of these single-phase SCPE samples in standard SBF and SBF modified with ALP.

### 3.2. Morphological changes of SCPEs in SBFs

**Figure 3.**
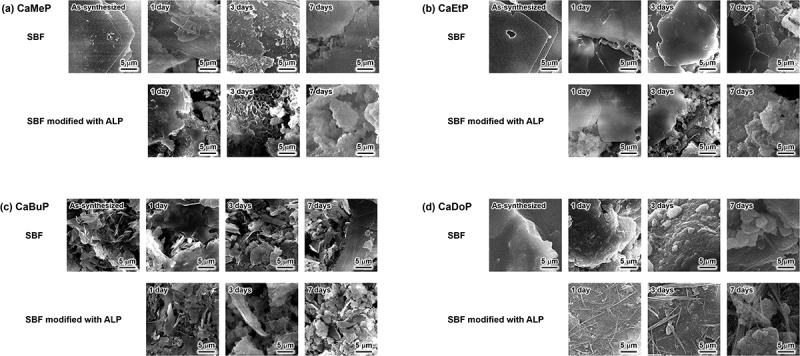
SEM images of (a) CaMeP, (b) CaEtP, (c) CaBuP and (d) CaDoP before and after soaking in standard SBF and SBF modified with ALP.

The morphologies of samples soaked in the standard SBF and SBF modified with ALP for up to 7 days are shown in Figure 3. As-synthesized CaMeP had a plate-like shape with particle sizes of 10–100 µm (Figure 3(a)). After soaking in standard SBF, no significant morphological changes of CaMeP were observed on day 1; however, small amounts of fine precipitates were observed on days 3 and 7. CaMeP soaked in the SBF modified with ALP showed fine precipitates formed on day 1. On day 3, precipitates with specific petal-like structures were observed and they disappeared on day 7. In addition, the original shape of CaMeP had almost disappeared at day 7, and aggregates of fine irregular-shaped precipitates were observed.

As-synthesized CaEtP consisted of plate-like crystals with sizes of 10–150 µm (Figure 3(b)). No significant morphological change of CaEtP was observed until 3 days after soaking in the standard SBF. After 7 days of soaking in the SBF, fine precipitates were observed on the large plate-like crystals. On the other hand, when CaEtP was soaked in the SBF modified with ALP, fine precipitates were observed after 1 day, and the amount of fine particles was increased. After CaEtP was soaked in the SBF modified with ALP for 7 days, no plate-like crystals were observed and aggregates of fine irregular-shaped precipitates were found.

As-synthesized CaBuP consisted of approximately 5 µm sized plate-like crystals (Figure 3(c)). No significant morphological change of CaBuP occurred in the standard SBF up to day 7. After 1 day of CaBuP soaking in the SBF modified with ALP, the morphology was not changed from that before soaking, although after 3 and 7 days of soaking, fine granular precipitates were observed along with the plate-like CaBuP crystals.

In the case of CaDoP (Figure 3(d)), the as-synthesized particles were irregular in shape, and hemispherical-shaped precipitates several micrometers in size were observed on the surface of the CaDoP particles after 3 and 7 days of soaking in the standard SBF. Fibrous precipitates were observed on the surface of CaDoP particles after 1 day of soaking in the SBF modified with ALP. The diameter of the fibrous crystals increased with increasing soaking period.

### 3.3. Crystalline phase changes of SCPEs in SBFs

**Figure 4.**
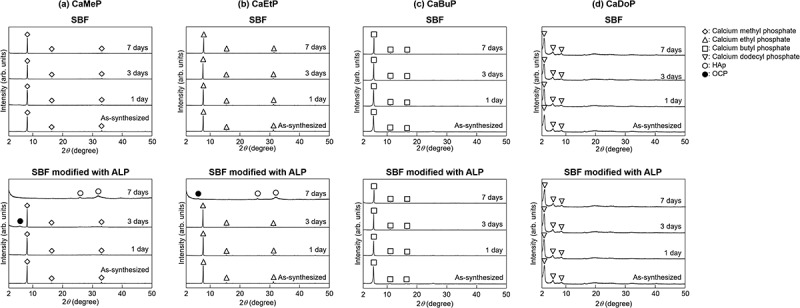
XRD patterns for as-synthesised CaMeP, CaEtP, CaBuP and CaDoP, and those after soaking in standard SBF and SBF modified with ALP.

XRD patterns for samples soaked in standard SBF and the SBF modified with ALP up to 7 days are shown in Figure 4. According to Figure 4(a), no crystalline phase change of CaMeP soaked in the standard SBF was detected. In contrast, octacalcium phosphate (OCP; Ca_8_(HPO_4_)_2_(PO_4_)_4_·5 H_2_O) was detected in the sample soaked in the SBF modified with ALP at 3 days. The powder diffraction file (PDF) #01-074-1301 was used to identify OCP. HAp was detected after 7 days of soaking, and no CaMeP or OCP was detected. The PDF #00-009-0432 was used to identify HAp. Although the peak derived from HAp appears to be weak, a magnified XRD pattern is shown in Figure S1 in the supplementary information to demonstrate that the peak intensity is sufficient to identify the crystalline phase. According to Figure 4(b), no crystalline phase change of CaEtP soaked in the standard SBF was detected. However, the sample soaked in the SBF modified with ALP revealed HAp and OCP at 7 days, and CaEtP was absent. A magnified XRD pattern for this sample is shown in Figure S2 in the supplementary information. No change in the crystalline phase of CaBuP soaked in the standard SBF was detected using XRD (Figure 4(c)). HAp was detected along with CaBuP after soaking in the SBF modified with ALP for 7 days. However, the peaks for HAp were very weak and barely observable. Therefore, magnified XRD patterns for CaBuP before and after soaking in the SBF modified with ALP at 7 days are shown in Figure S3 in the supplementary information. No change in the crystalline phase of CaDoP was detected by XRD measurements, regardless of ALP addition to the SBF (Figure 4(d)).

### 3.4. Changes in Ca and P concentrations, and pH of SBFs caused by soaking SCPEs

**Figure 5.**
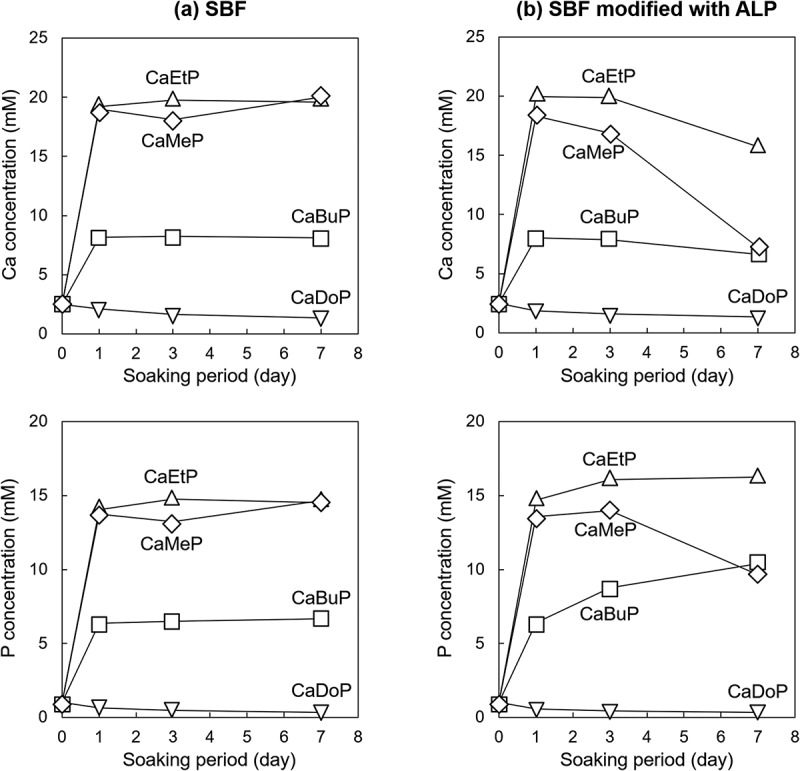
Time-dependent changes of Ca and P concentrations in (a) standard SBF and (b) SBF modified with ALP.

The Ca and P concentrations in the SBFs evaluated by ICP-AES measurements are shown in Figure 5. Note that the P concentration is the sum of the concentration of phosphoric acid and the concentration of phosphate esters. The Ca concentrations in the standard SBF with CaMeP, CaEtP, and CaBuP increased sharply and reached approximately 18 mM, 19 mM, and 8 mM on 1 day, respectively, and then remained almost constant until day 7 (Figure 5(a)). In addition, the P concentrations of the SBF with CaMeP, CaEtP, and CaBuP increased sharply and reached approximately 14 mM, 14 mM, and 7 mM on 1 day, after which they remained almost constant until day 7 (Figure 5(a)). In contrast, the Ca and P concentrations in the SBF with CaDoP decreased slightly over the 7 days (Figure 5(a)).

The Ca concentrations of the SBF modified with ALP, in which CaMeP and CaEtP were soaked, increased sharply, and reached approximately 18 mM and 20 mM on day 1, respectively, and then decreased to 8 mM and 17 mM at day 7, respectively (Figure 5(b)). In the case of CaBuP, the Ca concentrations of the SBF increased sharply and reached 8 mM on day 1, and then decreased to 7 mM at day 7 (Figure 5(b)). The P concentrations of the SBF with CaMeP increased to 14 mM on day 1 and this concentration was kept until day 3, and then decreased to 10 mM on day 7 (Figure 5(b)). The P concentrations of the SBF with CaEtP increased significantly to 15 mM on day 1, and then gradually increased to 17 mM on day 7 (Figure 5(b)). The P concentrations of the SBF with CaBuP increased to 7 mM on day 1, gradually increased to 9 mM on day 3, and then reached 10 mM on day 7 (Figure 5(b)). Similar to the case of the standard SBF, the Ca and P concentrations in the SBF modified with ALP in which CaDoP was soaked decreased slightly over 7 days (Figure 5(b)).

**Figure 6.**
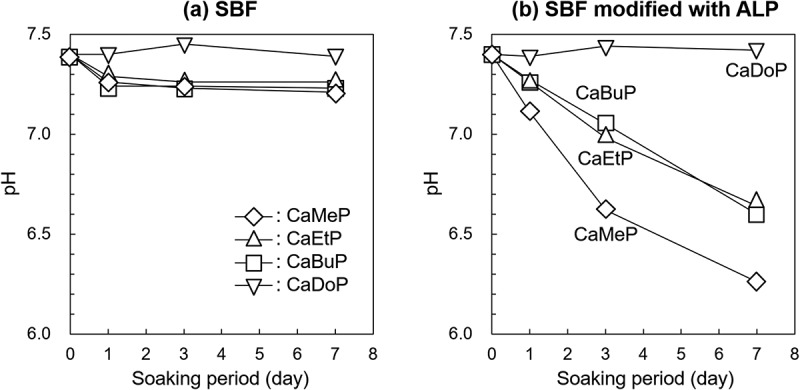
Time-dependent changes of pH in (a) standard SBF and (b) SBF modified with ALP.

Figure 6(a) shows the changes in pH of the SBFs due to soaking of the SCPEs. The pH of the standard SBF after soaking CaMeP, CaEtP, and CaBuP decreased slightly from 7.4 to 7.2–7.3 at day 1, and then remained constant until day 7. In the case of CaDoP, little change in the pH of the SBF occurred over 7 days. Figure 6(b) shows the changes in pH of the SBF modified with ALP due to soaking of the SCPEs. The pH of the SBF with CaMeP decreased from 7.4 to 7.1 on day 1, and then the pH continuously decreased and reached 6.3 at day 7. The pH of the SBF with CaEtP and CaBuP decreased to 7.2–7.3 at day 1, and then the pH continuously decreased to around 7.0 at day 3, and then finally reached 6.1–6.2 at day 7. In contrast to the other samples, the pH of the SBF with CaDoP remained constant at 7.4 for 7 days.

## 4. Discussion

### 4.1. Behaviour of SCPEs in SBF with and without ALP modification

According to Figures 3–6, the behaviour of CaMeP, CaEtP, and CaBuP in the SBF modified with ALP was completely different from that in the standard SBF without ALP modification. Precipitate formation on these SCPEs in the standard SBF was observed using SEM (Figure 3). However, the amount of precipitates formed in the SBF was so small that they could not be detected by XRD analysis (Figure 4). Considering that SBF is a supersaturated solution with respect to HAp, the precipitates are likely to be HAp and/or amorphous calcium phosphate. According to Figure 5(a), the Ca and P concentrations in the SBF increased after CaMeP, CaEtP, and CaBuP were soaked in it. This increase in the Ca^2+^ concentrations caused by dissolution of these SCPEs increased the degree of supersaturation with respect to HAp and promoted the precipitation of calcium phosphate. As an example, the reaction equation for the formation of HAp with a stoichiometric composition is

(1) $$10Ca^{2+}+6P{O_{4}}^{3-}+2OH^{-}\to Ca_{10}{\left(PO_{4}\right)}_{6}{\left(OH\right)}_{2}$$

The P concentration here is the sum of the phosphoric acid concentration, the phosphate ester concentration, and the concentration of related P-containing chemical species; therefore, it is unclear how much of an effect the increase in the P concentration had on the precipitation of calcium phosphates. To further elucidate the direct effect of the increase in phosphate ion activity on precipitate formation, it is thus necessary to quantitatively clarify the fraction of these P-containing chemical species in the SBF, namely phosphate esters, phosphate ions and complex ions including phosphate esters and phosphate ions.

**Figure 7.**
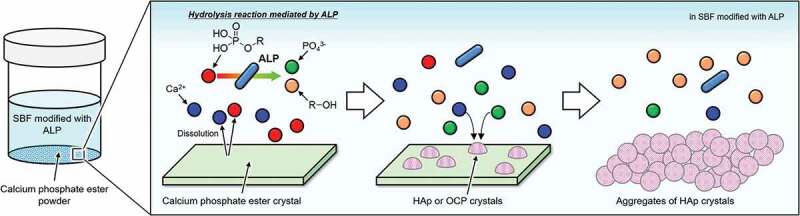
Schematic illustration of transformation of SCPEs in SBF modified with ALP.

The XRD patterns for CaMeP and CaEtP clearly indicated that these SCPEs were transformed into HAp, and HAp and OCP, respectively, after soaking in the SBF modified with ALP (Figure 4). The decrease in the Ca^2+^ concentration after 1 day (Figure 5(b)) and the decrease in the pH of the SBF (Figure 6(b)) suggest the formation of calcium phosphate, which supports the results of the XRD analysis. The transformation reaction of CaMeP and CaEtP did not proceed readily in the standard SBF; therefore, it is considered that ALP caused hydrolysis of the methyl- and ethyl phosphates, which led to the transformation of these SCPEs. In addition, with this transformation in crystalline phases, the plate-like crystals of CaMeP and CaEtP were changed into aggregates of fine particles (Figure 3(a,b)). Thus, it was determined that CaMeP and CaEtP can be transformed into calcium phosphate phases, such as HAp and OCP in the SBF containing human plasma levels of ALP. The transformation steps for SCPEs were (i) dissolution of SCPEs, (ii) hydrolysis of phosphate esters eluted from the SCPEs mediated by ALP, and (iii) calcium phosphate precipitation. A schematic illustration of the transformation sequence is shown in Figure 7. For CaBuP, the XRD patterns showed that a small amount of HAp was produced in SBF modified with ALP after 7 days (see Figure S3), and a decrease in the Ca^2+^ concentration after 1 day and a decrease in the pH of the SBF were also observed. Such behaviour was the same as for CaMeP and CaEtP; therefore, CaBuP was also transformed into HAp in the SBF modified with ALP by the same transformation process as that for CaMeP and CaEtP. However, the rate of the transformation reaction for CaBuP was clearly slower than that for CaMeP and CaEtP.

The behaviour of CaDoP in SBF with and without ALP modification was completely different from that of the other SCPEs. Table 1 and Figure 5 show that CaDoP had very low solubility compared to the other SCPEs. Therefore, formation of calcium phosphates due to an increase in the Ca^2+^ concentration by the dissolution of CaDoP is not desirable. However, hemispherical precipitates were formed on the surface of the plate-like CaDoP crystals in the standard SBF (Figure 3(d)). This hemispherical morphology is typical of the HAp produced in SBF [48], which suggests that CaDoP has the ability to form HAp in the standard SBF. In contrast, fibrous precipitates were formed in the SBF modified with ALP (Figure 3(d)), although the changes in the Ca and P concentrations, and the pH of the SBF with the sample soaking period were almost the same, regardless of the presence of ALP (Figures 5 and 6). Fibrous-shaped HAp and OCP were reported previously [49,50]; therefore, these precipitates are likely to be HAp and/or OCP, although these crystalline phases could not be detected by XRD because their amount was lower than the detection limit (Figure 4(d)). Based on the SEM observation that the morphology of the precipitates was different in the presence and absence of ALP (Figure 3(d)), ALP likely had an effect on the formation of precipitates in the ALP-modified SBF soaked with CaDoP. According to crystal growth theory, crystals with a morphology close to the equilibrium form, such as fibrous crystals, are formed under low supersaturation environments [51]. Therefore, supersaturation of the reaction field with respect to HAp and OCP was likely suppressed by the addition of ALP due to the reducing activity of calcium and/or phosphate ions.

The most important conclusion in this section is that CaMeP, CaEtP, and CaBuP were determined to be bioresponsive ceramics that respond to ALP. In addition, CaDoP may also be a bioresponsive ceramic.

### 4.2. Effects of phosphate ester alkyl groups on rate and rate-determining steps of for transformation in SBF modified with ALP

Insight into the transformation rate for the SCPEs was successfully obtained; larger alkyl groups in the phosphate ester resulted in a smaller transformation rate for the SCPEs in the SBF modified with ALP, as shown in Figure 4. The order of the transformation rates for the SCPEs seems to be closely related to their solubility (Table 1). Therefore, we next discuss the rate-determining step for the transformation reaction.

When regarding the transformation of SCPEs to calcium phosphate, it is necessary to consider the rate-determining steps, that is, the dissolution of SCPEs and the hydrolysis rate for phosphate esters mediated by ALP. The calcium phosphate formation rate is expected to be faster than these processes; therefore, they are not considered here. The rate-determining steps can be inferred from changes in the Ca and P concentrations in the SBF modified with ALP after day 1. The decreases in the Ca and P concentrations for CaMeP in SBF modified with ALP were almost identical after day 1 (Figure 5(b)). This suggests that the hydrolysis of methyl phosphate mediated by ALP occurred rapidly, that is, the rate-determining step for the HAp formation reaction is most likely the dissolution of CaMeP. For CaEtP, the Ca concentration decreased after 1 day, whereas the P concentration increased slightly (Figure 5(b)). The crystalline phases of calcium phosphate produced in the reaction system in this study were HAp (Ca/P = 1.67, stoichiometric composition) and OCP (Ca/P = 1.33), both of which are calcium-rich compounds. Therefore, it is difficult to determine the rate-determining step for the HAp and OCP formation reactions from CaEtP. For CaBuP, the Ca concentration gradually decreased after the first day, whereas the P concentration appreciably increased (Figure 5(b)). Butyl phosphate rather than phosphoric acid is likely responsible for this increase in the P concentration. Therefore, the rate-determining step for the transformation of CaBuP to HAp is most likely the hydrolysis of butyl phosphate mediated by ALP. Consequently, it can be concluded that when the number of carbon atoms in the linear alkyl group in the phosphate ester increases to 4 (i.e. butyl group), the rate-determining step for the transformation changes from dissolution of the SCPEs to hydrolysis of the phosphate ester with SBF containing human plasma levels of ALP.

In the case of CaDoP, the time-dependent changes of the Ca and P concentrations, and the pH were almost the same, regardless of the presence or absence of ALP in SBF (Figures 5(b) and 6(b)), although the morphology of the precipitates, which is likely a calcium phosphate phase, was different (Figure 3(d)). Therefore, it is difficult to determine the rate-determining step for the transformation of CaDoP to a calcium phosphate. In addition, precipitates were observed using SEM and the crystalline phases are likely to be HAp and/or OCP; however, there is insufficient evidence to confirm this. Therefore, further study is required to clarify the transformation reaction of CaDoP in the SBFs.

## 5. Conclusions

We investigated the behaviour of calcium salts of methyl phosphate, ethyl phosphate, butyl phosphate, and dodecyl phosphate in SBF with and without ALP modification. In the standard SBF, these SCPEs did not readily transform into a calcium phosphate phase, although small amounts of precipitates, which are likely a calcium phosphate phase formed from the SBF, were observed. On the other hand, in the SBF modified with ALP, CaMeP, CaEtP, and CaBuP were transformed into HAp and OCP, and these SCPEs are thus considered to be categorised as bioresponsive ceramics that respond to ALP. CaDoP is also most likely a bioresponsive ceramic, although no vigorous response was detected due to its very low solubility. The main finding was that a larger linear alkyl group in the phosphate ester resulted in a smaller transformation rate for the SCPEs in the SBF. The rate-determining step for the transformation reaction of the SCPEs were also found to change from dissolution of the SCPEs to hydrolysis of the phosphate esters mediated by ALP with increasing size of the phosphate ester alkyl groups in the SCPEs. We consider these findings a worthwhile contribution to the design and development of novel bioresponsive ceramic biomaterials.

## Supplementary Material

Supplemental Material
