# Supplementary material for: Transformation behaviour of salts composed of calcium ions and phosphate esters with different linear alkyl chain structures in a simulated body fluid modified with alkaline phosphatase
Source: Sci Technol Adv Mater. 2022 May 30;23(1):341–51. doi: 10.1080/14686996.2022.2074801 (PMC9176335; doi:10.1080/14686996.2022.2074801)
Supplement: Supplemental Material [file TSTA_A_2074801_SM5154.pdf]

Supplementary information

Transformation behaviour of salts composed of calcium ions and phosphate esters with different alkyl chain structures in a simulated body fluid modified with alkaline phosphatase

Taishi Yokoi,[a]* Akiyoshi Mio,[b] Jin Nakamura,[b,#] Ayae Sugawara-Narutaki,[b] Masakazu Kawashita[a] and Chikara Ohtsuki[b]

[a] *Institute of Biomaterials and Bioengineering, Tokyo Medical and Dental University (TMDU), 2-3-10 Kanda-Surugadai, Chiyoda-ku, Tokyo 101-0062, Japan.*

[b] *Graduate School of Engineering, Nagoya University, Furo-cho, Chikusa-ku, Nagoya 464-8603, Japan.*

[#] *Present address: Graduate School of Life Science and Systems Engineering, Kyushu Institute of Technology, 2-4 Hibikino, Wakamatsu-ku, Kitakyushu-shi, Fukuoka 808-0196, Japan.*

E-mail for the corresponding author: yokoi.taishi.bcr@tmd.ac.jp

Preparation of SBF

Simulated body fluid (SBF) was prepared as follows: 700 cm^3 of ultrapure water was added to a 1000 cm^3 glass beaker and stirred with a magnetic stirrer. The reagent-grade chemicals given in Table S1 were then dissolved in the order listed. Each reagent was allowed to completely dissolve before the addition of the next reagent. The solution was maintained at 36.5 °C and the pH was adjusted to 7.4 by the addition of 1.0 $mol{\cdot}dm^{-3}$ hydrochloric acid. After pH adjustment, the solution was transferred to a volumetric flask and ultrapure water was added to adjust the total volume of the solution to 1000 cm^3. All reagents shown in Table S1, except for the 1.0 $mol{\cdot}dm^{-3}$ hydrochloric acid solution, were purchased from Nacalai Tesque, Inc., Kyoto, Japan. The 1.0 $mol{\cdot}dm^{-3}$ hydrochloric acid solution was prepared by dilution of a 35 mass% hydrochloric acid solution (HCl, Wako Pure Chemical Industries Ltd., Osaka, Japan).

Table S1 Reagents used to prepare 1000 cm^3 of SBF.

Order	Reagent	Amount
1	NaCl	7.996 g
2	$NaHCO_3$	0.350 g
3	KCl	0.224 g
4	$K_2HPO_4{\cdot}3H_2O$	0.228 g
5	$MgCl_2{\cdot}6H_2O$	0.305 g
6	1.0 $mol{\cdot}dm^{-3}$ HCl	40 cm^3
7	$CaCl_2$	0.278 g
8	Na_2SO_4	0.071 g
9	$(CH_2OH)_3CNH_2$	6.057 g

Figures

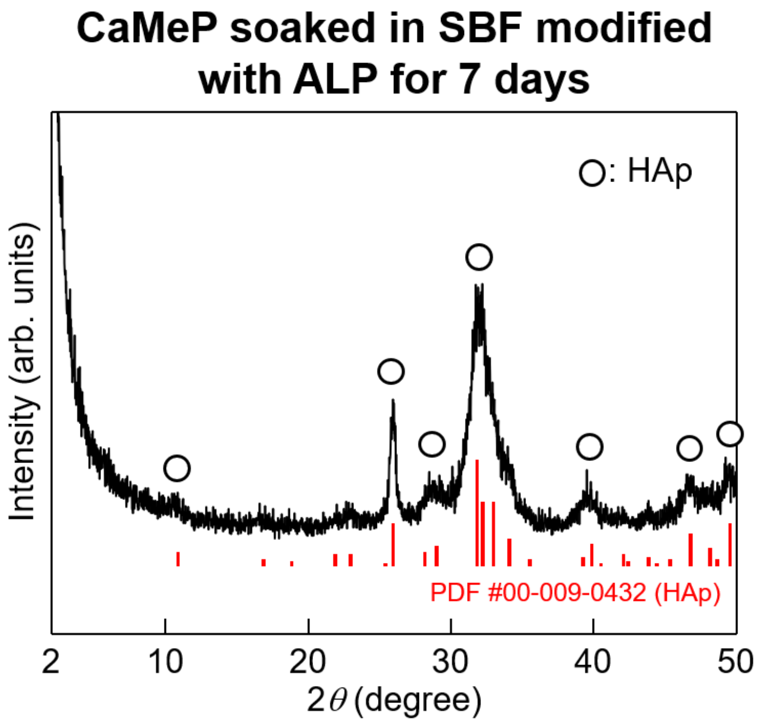

Figure S1. Magnified XRD pattern for CaMeP soaked in SBF modified with ALP for 7 days.

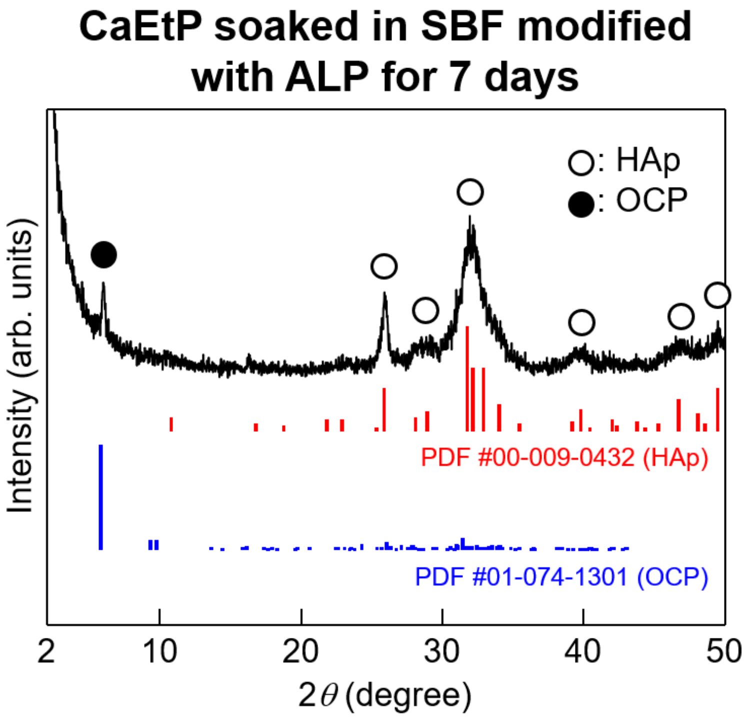

Figure S2. Magnified XRD pattern for CaEtP soaked in SBF modified with ALP for 7 days.

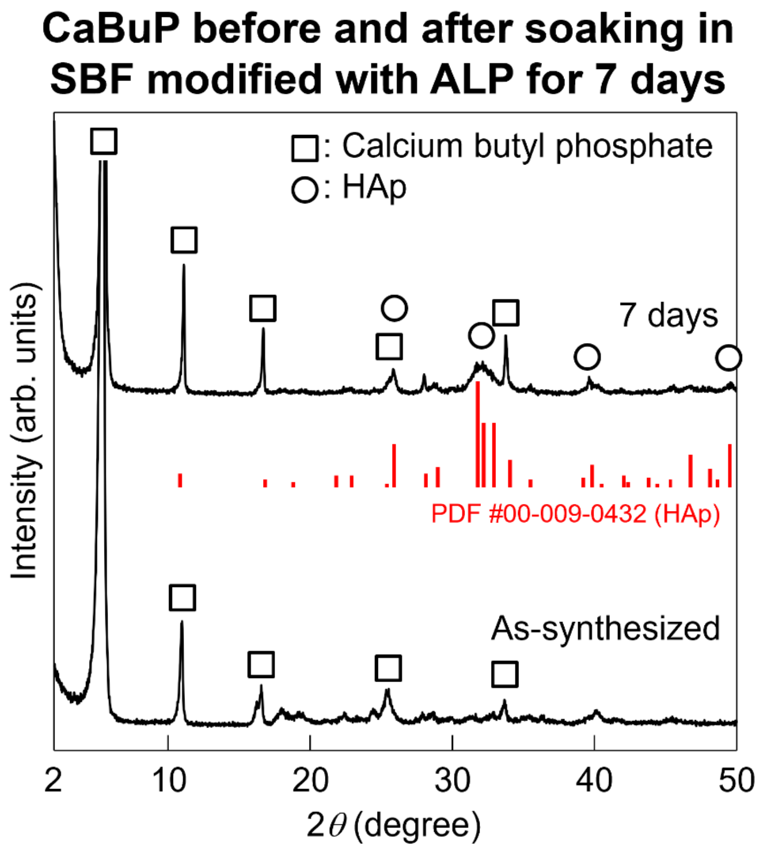

Figure S3. Magnified XRD patterns for CaBuP before and after soaking in SBF modified with ALP for 7 days.
